# Rhein Inhibits Autophagy in Rat Renal Tubular Cells by Regulation of AMPK/mTOR Signaling

**DOI:** 10.1038/srep43790

**Published:** 2017-03-02

**Authors:** Yue Tu, Liubao Gu, Diping Chen, Wei Wu, Hong Liu, Hao Hu, Yigang Wan, Wei Sun

**Affiliations:** 1 Department of Nephrology, The Affiliated Hospital of Nanjing University of Chinese Medicine, Nanjing, China; 2 Department of TCM Health Preservation, Second Clinic Medical School, Nanjing University of Chinese Medicine, Nanjng, China; 3 Center for Diabetes Care, Education and Research, Jiangsu Province Institute of Geriatrics, Nanjing, China; 4 Department of Traditional Chinese Medicine, Nanjing Drum Tower Hospital, The Affiliated Hospital of Nanjing University Medical School, Nanjing, China; 5 First Clinic Medical School, Nanjing University of Chinese Medicine, Nanjng, China; 6 Department of Nephrology, Huanggang Hospital of Traditional Chinese Medicine, Huanggang, China

## Abstract

Rhubarb and its bioactive component rhein are frequently used for the treatment of chronic kidney diseases (CKD) in eastern Asia countries. However, the potential therapeutic mechanism remains unclear. Autophagy plays an important role in CKD. However, there were some important related issues that remained unresolved in the role of autophagy in CKD and treatment by rhubarb and rhein. We designed a number of experiments to examine whether rhubarb may reduce renal fibrosis and autophagy in rats with adenine (Ade)-induced renal tubular injury, and whether rhein could affect autophagic pathways in rat renal tubular cells. We found that, autophagic activation accompanied with renal fibrosis in rats with Ade-induced renal tubular injury, and both autophagy and renal fibrosis were attenuated by rhubarb. In addition, we observed that rhein could inhibit autophagy through regulating the key molecules in the AMPK-dependent mTOR signaling pathways, as well as the Erk and p38 MAPKs signaling pathways. These findings may partly explain the therapeutic mechanisms of rhubarb and rhein in treating CKD patients in clinic, and further suggest that targeting autophagy and related signaling pathways may provide new strategies for the treatment of renal fibrosis in CKD.

Traditional Chinese herbal medications (TCHMs) are frequently used for the treatment of chronic kidney diseases (CKD) in eastern Asia countries^1^. A wealth of evidence has shown that TCHMs could improve renal dysfunction^2^,^3^,^4^; among them rhubarb, (also known as Radix et Rhizoma Rhei, and as Da huang in China), is derived from the root of *Rheum palmatum* (Fig. 1A), and it has been demonstrated to ameliorate renal dysfunction and the glomerular filtration rate (GFR) in patients with CKD stage 3 and 4 in clinic^5^. In addition, rhubarb has been found to attenuate renal fibrosis in rats with chronic renal failure and subtotal nephrectomy^6^. Rhein (the chemical and molecular structures are shown in Fig. 1B), as a bioactive component of rhubarb, has also been shown to alleviate renal interstitial fibrosis and inhibit the expression of transforming growth factor (TGF)-β1, fibronectin and alpha-smooth muscle actin (α-SM actin) in unilateral ureteral obstruction (UUO) kidneys^7^. Despite these studies, the potential therapeutic mechanisms of rhubarb and rhein on renal fibrosis remain unclear. In our previous study, we found that a specific Chinese herbal compound prescription containing rhubarb (Dahuang Fuzi Decoction, DFD) could improve adenine (Ade)-induced renal fibrosis and renal tubular cell damage *in vivo*^8^,^9^. Furthermore, Livingston *et al*. reported that persistent activation of autophagy in renal proximal tubules could promote renal interstitial fibrosis^10^. These results suggested that the beneficial effect of rhubarb and rhein on renal fibrosis in CKD might have a close relationship with autophagy in renal tubular cells.

Autophagy plays an important role in regulating cell death^11^. Autophagy is a self-degrading process, which can be activated under certain circumstances including starvation, hypoxia, ischemia/reperfusion, stress, and infection^12^,^13^. Depending on the experimental conditions, autophagy may protect against cell death (protective role) or lead to cell injury (detrimental role)^14^. In hepatic stellate cells, autophagy can break down lipids to fuel the activation of these cells to promote liver fibrosis^15^,^16^. In the kidney, autophagy has been suggested to induce tubular atrophy and decomposition to promote fibrosis in the UUO model^17^,^18^. It has been reported that in rats with ischemia/reperfusion-induced renal dysfunction, the expressions of the autophagic markers microtubule-associated protein 1 light chain 3 (LC3) and beclin1 were increased together with apoptosis^19^. In the human renal proximal tubular epithelial cell line (HK-2), autophagy inhibitors were shown to suppress H_2_O_2_-induced cell death, suggesting that autophagy might contribute to cell death during kidney impairment^20^. In contrast, in some pathological conditions, autophagic activity plays a protective role in renal injury. In rats that had been treated with puromycin aminonucleoside (PAN), the inhibition of autophagy with 3-methyladenine (3-MA) or chloroquine resulted in earlier podocytopathies, whereas rapamycin-mediated stimulation of autophagy led to less renal injury^21^. Overall, the role of autophagy in CKD is controversial.

Up to present, there are still some important issues unresolved in the role of autophagy in CKD treated by rhubarb and rhein, for example, whether rhubarb can ameliorate renal fibrosis through regulation of autophagy, and if yes, what are the underlying mechanisms involved. To address these important issues, we designed a number of *in vivo* and *in vitro* experiments to examine the hypothesis that rhubarb may reduce renal fibrosis and autophagy in rats with Ade-induced renal tubular injury, and that rhein may affect autophagic pathways in NRK-52E cells. Results in agreement with this hypothesis would suggest that the suppression of autophagy is protective in CKD.

## Results

### Renal Fibrosis Aggravated by Autophagy is Ameliorated by Rhubarb *in Vivo*

**Figure 1:**
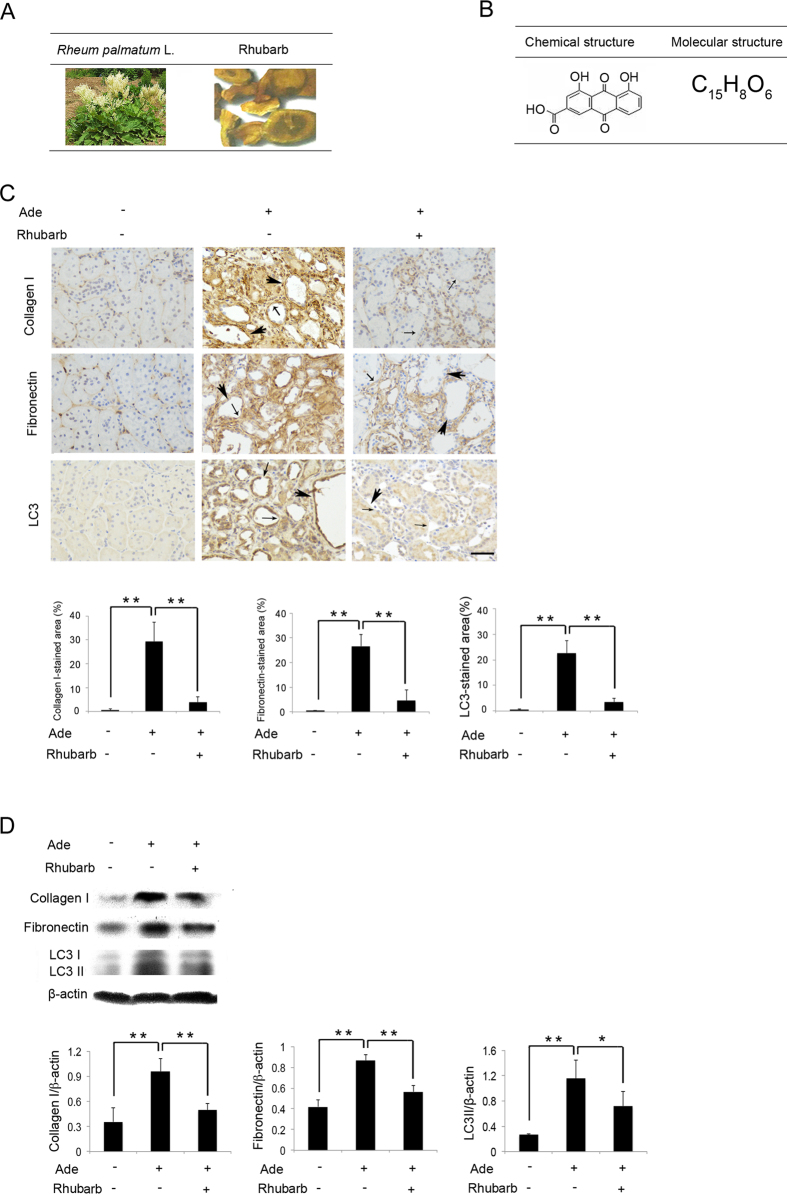
Renal Fibrosis Aggravated by Autophagy is Ameliorated by Rhubarb in Vivo. (**A**) The fresh plant of *Rheum palmatum* L. and the processed decoction pieces of rhubarb (Radix et Rhizoma Rhei). (**B**) Chemical and molecular structures of rhein (A bioactive component of rhubarb). (**C**) Immunohistochemical staining of collagen I, fibronectin and LC3, and the percentage of the positively stained areas of collagen I, fibronectin and LC3. Scale bar = 20 μm. The thick and short arrows show dilated tubules, the fine and long arrows show reduced tubular epithelial cells, and the dark brown areas show increased renal interstitial areas. (**D**) Western blot analysis of collagen I, fibronectin and LC3 I/II. Data are expressed as mean ± SD, *P < 0.05, **P < 0.01. Abbreviation: Ade, adenine.

To investigate whether rhubarb can inhibit renal fibrosis and interfere with autophagic activity in rats with Ade-induced renal tubular injury, we examined the changes in immunohistochemical staining of the fibrotic markers collagen type I (collagen I) and fibronectin, as well as autophagic marker LC3 in the kidney. Compared to the control group, we observed obvious pathological changes including dilated tubules, reduced tubular epithelial cells and increased renal interstitial areas (Fig. 1C); in addition, the positively stained areas of collagen I, fibronectin, and LC3 in the kidney were increased significantly in the Ade group. The positively stained areas and pathological changes were decreased significantly in the Ade + rhubarb group compared to that in the Ade group. Consistent with these results, the protein expression of collagen I, fibronectin, and LC3 II (autophagic active marker) in the kidney were markedly upregulated in the Ade group, and downregulated significantly in the Ade + rhubarb group (Fig. 1D). In brief, these results indicated that autophagic activation accompanied with renal fibrosis, and rhubarb ameliorated autophagy and attenuated renal fibrosis in rats with Ade-induced renal tubular injury.

### Autophagy is Inhibited by Rhein *in Vitro*

**Figure 2:**
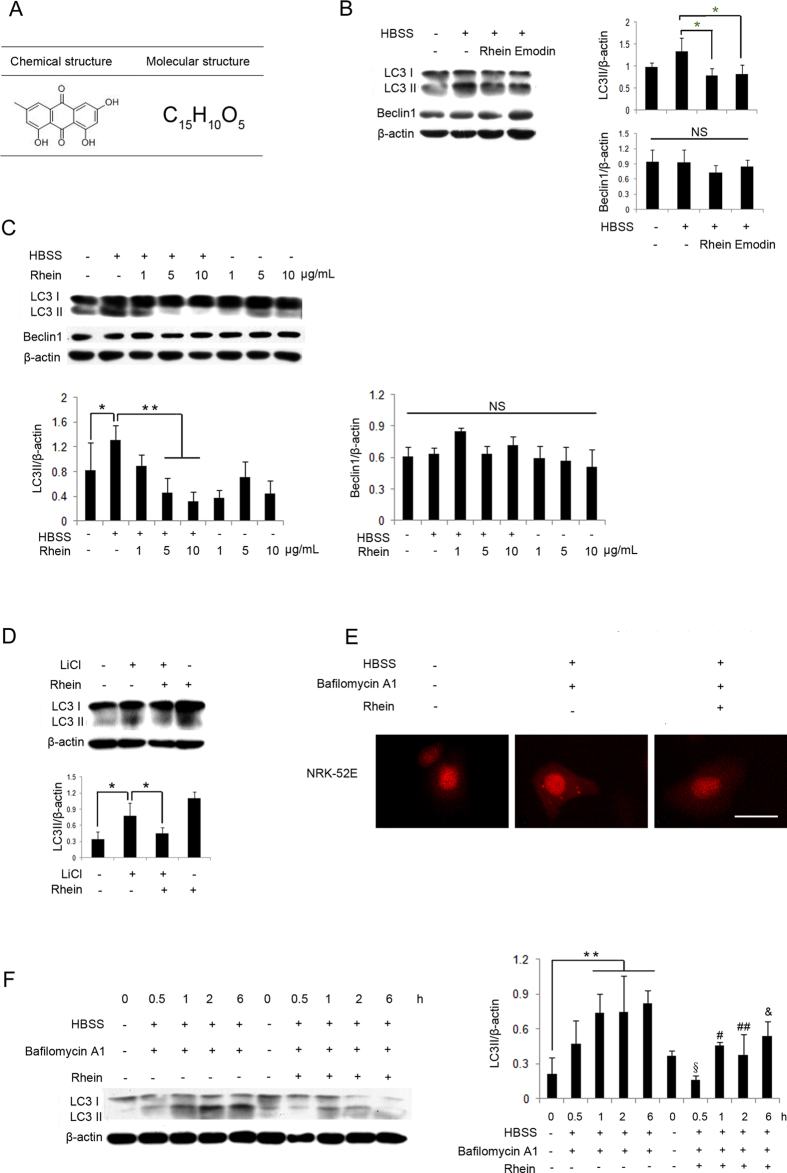
Autophagy is Inhibited by Rhein in Vitro. (**A**) Chemical and molecular structures of emodin (A bioactive component of rhubarb). (**B**) NRK-52E cells were treated with rhein (C_15_H_8_O_6_) 1 μg/ml and emodin (C_15_H_10_O_5_) 10 μM with or without HBSS for 1 hour and subjected to western blot analysis of LC3 I/II and beclin1. (**C**) NRK-52E cells were exposed to rhein 1, 5, and 10 μg/ml with or without HBSS for 1 hour and subjected to western blot analysis of LC3 I/II and beclin1. (**D**) NRK-52E cells were treated with rhein 5 μg/ml with or without LiCl 10 mM for 1 hour and subjected to western blot analysis of LC3 I/II. (**E**) NRK-52E cells were transfected with mRFP-LC3 and treated with rhein 5 μg/ml with or without HBSS and bafilomycin A1 10 nM for 2 hours and subjected to fluorescence microscopy. Scale bar = 5 μm. (**F**) NRK-52E cells were exposed to HBSS and bafilomycin A1 10 nM with or without rhein 1 μg/ml for 0, 0.5, 1, 2 and 6 hours, and subjected to western blot analysis of LC3 I/II. Data are expressed as mean ± SD, *P < 0.05, ^**^P < 0.01, ^§^*P* < 0.05 vs. co-treatment of HBSS and bafilomycin A1 at 0.5 hour, ^#^*P* < 0.05 vs. co-treatment of HBSS and bafilomycin A1 at 1 hour, ^##^*P* < 0.01 vs. co-treatment of HBSS and bafilomycin A1 at 2 h, ^&^*P* < 0.05 vs. co-treatment of HBSS and bafilomycin A1 at 6 hours, ^&&^*P* < 0.01 vs. co-treatment of HBSS and bafilomycin A1 at 6 hours. NS, not statistically significant. Abbreviation: HBSS, Hank's balanced salt solution; LiCl, lithium chloride.

A previous study has shown that autophagy can be induced by starvation^22^. As rhein and emodin (chemical and molecular structures are shown in Fig. 2A) are 2 major bioactive components of rhubarb, we treated NRK-52E cells incubated with Hank’s balanced salt solution (HBSS) with or without rhein or emodin to examine whether they can affect HBSS-induced autophagy. As shown in Fig. 2B, HBSS significantly induced LC3 conversion (LC3 I to LC3 II) in NRK-52E cells. This induction was markedly suppressed by the treatment of rhein and emodin. The expression of beclin1 was not noticeably changed in each group (Fig. 2B). HBSS-induced LC3 conversion was suppressed by rhein in a dose-dependent manner (Fig. 2C). To confirm the suppressive effect of rhein on autophagy, we tested the level of LC3 conversion treated by rhein in lithium chloride (LiCl)-induced autophagy^23^. We found that LC3 conversion can also be induced by LiCl in NRK-52E cells and suppressed by rhein (Fig. 2D). Intriguingly, in Fig. 2C and D, rhein alone can induce autophagy in NRK-52E cells, indicating that rhein exerts anti-autophagic effect only in pathological situation.

The green fluorescent protein (GFP)-LC3 or red fluorescent protein (RFP)-LC3 labeling method is often recommended as an essential approach to test the activity of autophagy, because testing LC3 conversion at the protein level alone has certain limitations^24^. Thus, NRK-52E cells were transiently transfected with a plasmid expressing pmRFP-tagged LC3 and then exposed to rhein. As shown in Fig. 2E, under nutrient-rich conditions, fluorescence was distributed throughout the cytoplasm, and a few punctate dots appeared in NRK-52E cells. As expected, an increased number of punctate structures were observed by the induction of starvation. In contrast, following the treatment with rhein, the increase in punctate dots was noticeably reduced. The quantification of LC3 punctas in 3 groups was shown in Supplementary Information Figure S1A. HBSS and bafilomycin A1 significantly increased LC3 punctas, which was reduced notably by treatment with rhein.

To test whether rhein indeed inhibits autophagic activity, the LC3 turnover assay was carried out^25^. Bafilomycin A1, an inhibitor of vacuolar H + -ATPase, can block autophagosome-lysosome fusion and diminish LC3 II degradation^26^. Figure 2F showed that the reduced level of LC3 II in rhein-treated cells was not affected by incubation with bafilomycin A1, indicating that the suppressive effect of rhein was not medicated by increased LC3 II degradation. In short, these results confirmed that rhein inhibited autophagy in NRK-52E cells.

### Autophagic Activity is inhibited by Rhein through mTOR Signaling

**Figure 3:**
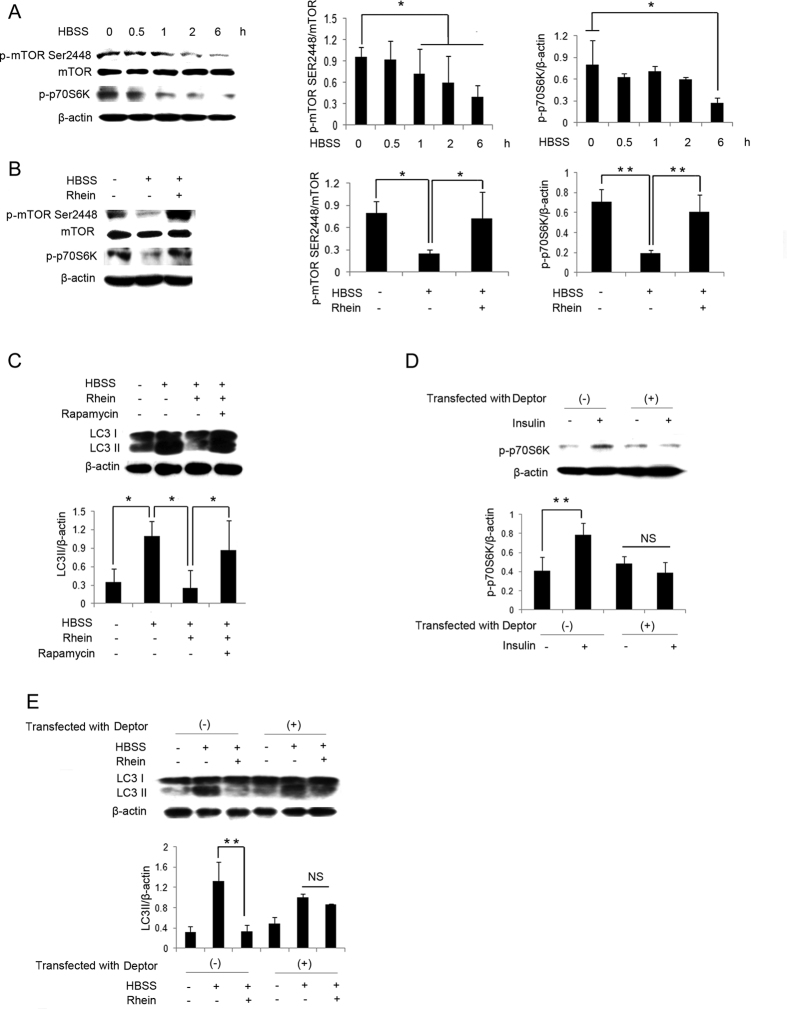
Autophagic Activity is inhibited by Rhein through mTOR Signaling. (**A**) NRK-52E cells were treated with HBSS for 0, 0.5, 1, 2 and 6 hours and subjected to western blot analysis of the phosphorylation of mTOR Ser2448 (p-mTOR Ser2448), mTOR and the phosphorylation of p70S6K (p-p70S6K). (**B**) NRK-52E cells were treated with HBSS with or without rhein 5 μg/ml for 6 hours and subjected to western blot analysis of p-mTOR Ser2448, mTOR and p-p70S6K. (**C**) NRK-52E cells were exposed to HBSS and rhein 5 μg/ml with or without rapamycin 100 mM for 1 hour and subjected to western blot analysis of LC3 I/II. (**D**) NRK-52E cells transfected with or without Deptor were exposed to insulin 10 μg/ml for 0.5 hour and subjected to western blot analysis of p-p70S6K. (**E**) NRK-52E cells transfected with or without Deptor were exposed to HBSS with or without rhein 5 μg/ml for 1 hour and subjected to western blot analysis of LC3 I/II. Data are expressed as mean ± SD, ^*^P < 0.05, ^**^P < 0.01, ^#^*P* < 0.05 vs. co-treatment of HBSS and rhein without rapamycin, NS, not statistically significant.

One of the key regulatory mechanisms of autophagy is mammalian target of rapamycin (mTOR) signaling, which has been identified as a suppressor of autophagic activity at the initiation of the vesicular double membrane formation^8^. Thus, the levels of phosphorylation of mTOR and its downstream substrate p70S6 kinase (p70S6K) were tested using immunoblotting. As shown in Fig. 3A, the phosphorylation of both mTOR Ser2448 and p70S6K were decreased in NRK-52E cells following starvation in a time-dependent manner. The downregulation of mTOR and p70S6K phosphorylation recovered following treatment with rhein (Fig. 3B).

To affirm the role of mTOR signaling in the suppressive effect of rhein on autophagic activity, we used rapamycin a specific inhibitor of mTOR activation^27^. As indicated in Fig. 3C, the decrease in LC3 conversion by rhein was reversed by rapamycin. The critical role of mTOR was further confirmed by the overexpression of Deptor, which specifically inhibits the activation of mTOR signaling through direct binding to both mTORC1 and mTORC2^28^. Transfection with Deptor effectively suppressed mTOR activity, because insulin-induced phosphorylation of p70S6K in NRK-52E cells was substantially diminished as shown in Fig. 3D. Consequently, in cells transfected with Deptor, the suppressive effect of rhein on LC3 II conversion was also abolished (Fig. 3E). In sum, these results proved that rhein inhibited autophagic activity through mTOR signaling in NRK-52E cells.

### Autophagic Activity is Inhibited by Rhein through Upstream Akt-independent and AMPK-dependent Signaling Pathways

**Figure 4:**
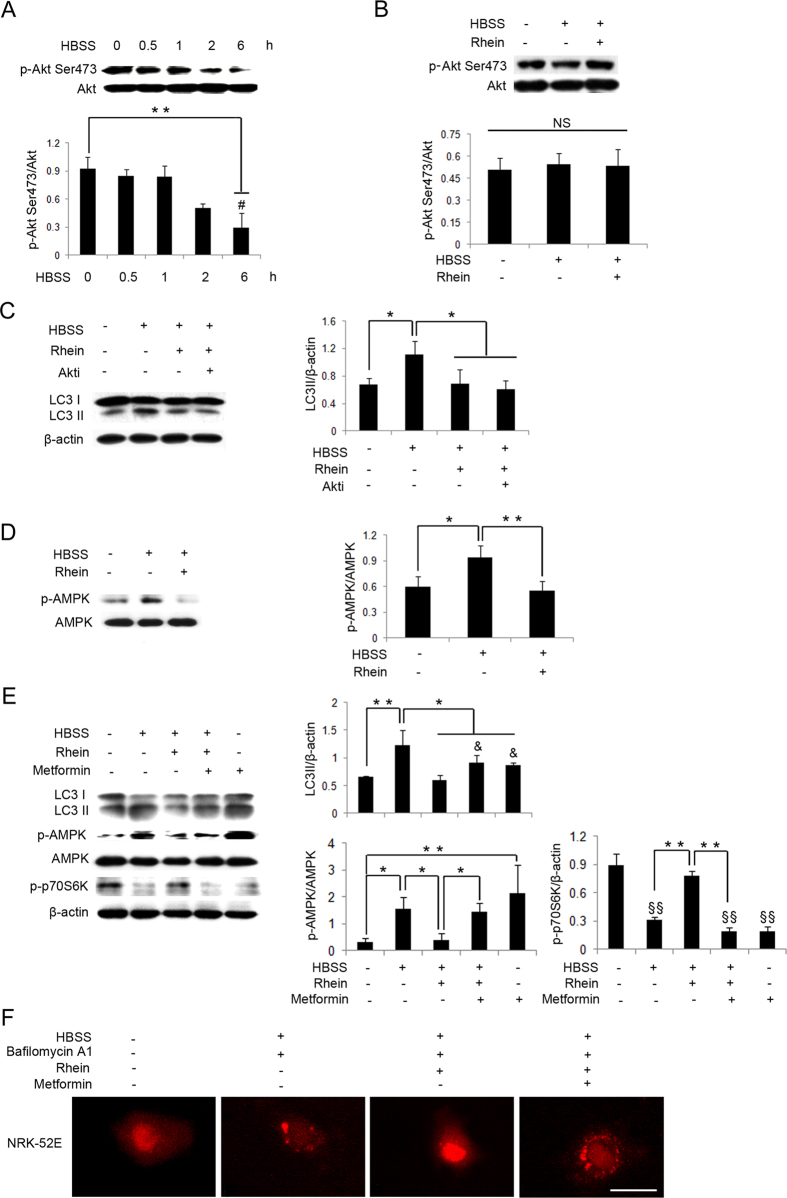
Autophagic Activity is Inhibited by Rhein through Upstream Akt-independent and AMPK-dependent Signaling Pathways. (**A**) NRK-52E cells were treated with HBSS for 0, 0.5, 1, 2 and 6 hours and subjected to western blot analysis of the phosphorylation of Akt Ser473 of (p-Akt Ser473) and Akt. (**B**) NRK-52E cells were exposed to HBSS with or without rhein 5 μg/ml for 1 hour and subjected to western blot analysis of p-Akt Ser473 and Akt. (**C**) NRK-52E cells were exposed to HBSS and rhein 5 μg/ml with or without Akti 10 μM for 1 hour and subjected to western blot analysis of LC3 I/II. (**D**) NRK-52E cells were exposed to HBSS with or without rhein 5 μg/ml for 2 hours and subjected to western blot analysis of the phosphorylation of AMPK (p-AMPK) and AMPK. (**E**) NRK-52E cells were exposed to HBSS and rhein 5 μg/ml with or without metformin (AMPK activator) 4 mM for 2 hours and subjected to LC3 I/II, p-AMPK, AMPK and p-p70S6K. (**F**) NRK-52E cells were transfected with mRFP-LC3 and treated with HBSS, bafilomycin A1 10 nM and rhein 5 μg/ml with or without metformin 4 mM for 2 hours and subjected to fluorescence microscopy. Scale bar = 5 μm. Data are expressed as mean ± SD, ^*^P < 0.05, ^**^P < 0.01, ^#^*P* < 0.05 vs. treatment of HBSS at 2 hours, ^&^*P* < 0.05 vs. co-treatment of HBSS and rhein, ^§§^*P* < 0.01 vs. control, NS, not statistically significant.

Phosphatidylinositol 3-kinase (PI3K)/serine-threonine kinase (Akt)/mTOR signaling pathway is the classical upstream pathway in regulating autophagy^29^. The phosphorylation of Akt was also decreased in a time-dependent manner following starvation (Fig. 4A), in parallel to the inhibition of mTOR activity. However, the treatment with rhein did not significantly affect the phosphorylation of Akt in NRK-52E cells exposed to HBSS (Fig. 4B). Moreover, Akti, an Akt inhibitor, also had an additional effect on the suppression of LC3 conversion by rhein in NRK-52E cells exposed to HBSS (Fig. 4C).

Adenosine monophosphate activated protein kinase (AMPK) is another important upstream molecule in the regulation of mTOR activity^30^, and we examined whether rhein affected mTOR signaling through the AMPK pathway. In this study, starvation induced the phosphorylation of AMPK, which was decreased by the co-treatment of rhein (Fig. 4D). Then we tested the central role of AMPK by using metformin, the commonly used AMPK activator^31^. Metformin restored the phosphorylation of AMPK suppressed by rhein. At the same time, metformin not only reduced the phosphorylation of p-p70S6K, but also increased LC3 conversion in NRK-52E cells exposed to HBSS and rhein (Fig. 4E). Moreover, fluorescence microscopy revealed that metformin increased punctate structures in mRFP-LC3 transfected NRK-52E cells treated with HBSS and rhein (Fig. 4F). The quantification of LC3 punctas in 4 groups was shown in Supplementary Information Figure S1B. The reduced LC3 punctas in HBSS, bafilomycin A1 and rhein group were reversely significantly increased by incubation with metformin. Therefore, these results showed that rhein inhibited autophagic activity through upstream Akt-independent and AMPK-dependent signaling pathways in NRK-52E cells.

### Autophagic Activity is Inhibited by Rhein through the MAPKs Signaling Pathways

**Figure 5:**
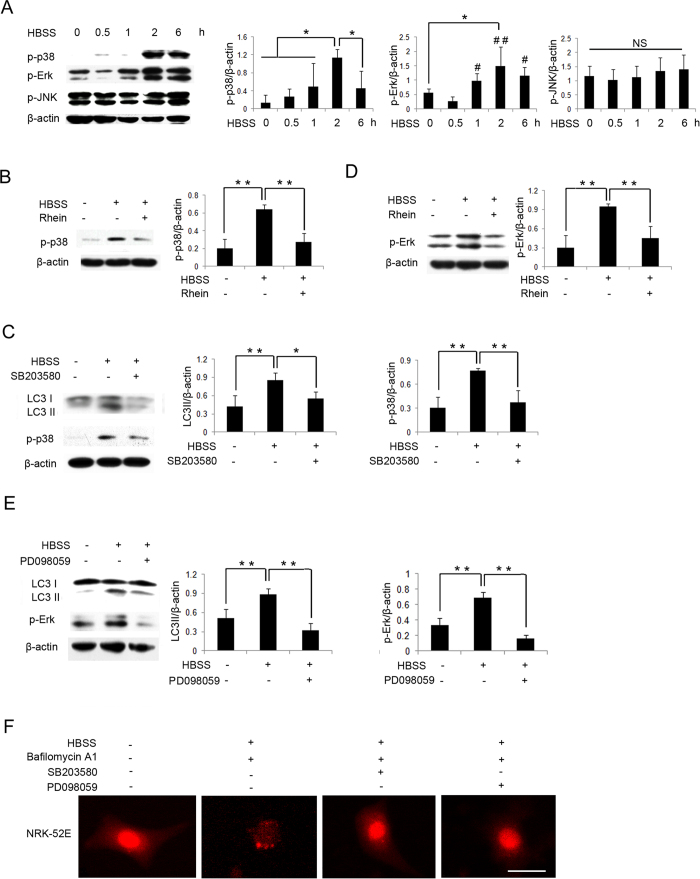
Autophagic Activity is Inhibited by Rhein through the MAPKs Signaling Pathways. (**A**) NRK-52E cells were treated with HBSS for 0, 0.5, 1, 2 and 6 hours and subjected to western blot analysis of the phosphorylation of p38 (p-p38), Erk (p-Erk) and JNK (p-JNK). (**B**) NRK-52E cells were exposed to HBSS with or without rhein 5 μg/ml for 6 hours and subjected to western blot analysis of p-p38. (**C**) NRK-52E cells were exposed to HBSS with or without SB203580 (a p38 inhibitor) 10 μM for 1 hour and subjected to western blot analysis of LC3 I/II and p-p38. (**D**) NRK-52E cells were exposed to HBSS with or without rhein 5 μg/ml for 6 hours and subjected to western blot analysis of p-Erk. (**E**) NRK-52E cells were exposed to HBSS with or without PD098059 (a p-Erk inhibitor) 50 μM for 1 hour and subjected to western blot analysis of LC3 I/II and p-Erk. (**F**) NRK-52E cells were transfected with mRFP-LC3 and treated with HBSS and bafilomycin A1 10 nM with or without SB203580 10 μM or PD098059 50 μM for 2 hours and subjected to fluorescence microscopy. Scale bar = 5 μm. Data are expressed as mean ± SD, ^*^P < 0.05, ^**^P < 0.01, NS, not statistically significant.

Mitogen-activated protein kinases (MAPKs) signaling pathways have been identified as the regulators of autophagy^7^. As shown in Fig. 5A, the phosphorylation of p38 and extracellular signal-regulated kinase (Erk) were induced in NRK-52E cells following starvation, in a time-dependent manner, whereas the phosphorylation of c-Jun N-terminal kinase (JNK) did not notably change (Fig. 5A). Rhein suppressed the phosphorylation of p38 and Erk induced by starvation (Fig. 5B and D). In addition, both the p38-MAPK inhibitor SB203580 and the Erk inhibitor PD098059 decreased LC3 conversion (Fig. 5C and E) and autophagic punctate structures (Fig. 5F). These results showed that rhein also inhibited autophagic activity through the p38 and Erk MAPKs signaling pathways.

## Discussion

**Figure 6:**
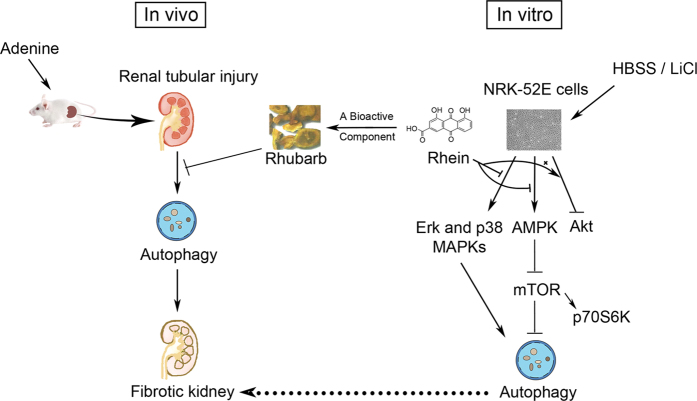
The Therapeutic Mechanisms of Rhubarb and Rhein in the Inhibition of Autophagy. Rhubarb suppressed renal fibrosis in rats with Ade-induced renal tubular injury by inhibiting autophagy. Rhein inhibited HBSS/LiCl-induced autophagy in NRK-52E cells. The suppressive effect of rhein on autophagy results from regulating the AMPK-dependent mTOR, the Erk and p38 MAPKs, and the Akt-independent signaling pathways.

In the present study, we demonstrated that autophagic activation accompanied with renal fibrosis, and that rhubarb could ameliorate autophagy and attenuate renal fibrosis in rats with Ade-induced renal tubular injury. Rhein, as a bioactive component of rhubarb, could inhibit autophagy through the Akt-independent and AMPK-dependent mTOR signaling pathways, and the Erk and p38 MAPKs signaling pathways were also involved in the suppressive effect of rhein on autophagy (Fig. 6).

Autophagy triggers kidney injury in some contexts, underscoring its nature as a double-edged sword that could be either protective or injurious depending on the cellular environment, the nature and intensity of the stimulus, and the level of autophagy^19^,^20^,^32^,^33^. Feeding Ade to rats resulted in marked tubular and interstitial injuries and metabolic abnormalities, characterized by tubular atrophy, renal dysfunction and proteinuria, which resemble chronic renal failure (CRF) in humans^34^. Our previous study proved that administering Ade could generate the model of rats with renal failure and renal fibrosis^8^. It might be supposed that autophagy would play a protective role against cell death rather than lead to cell lesion. Our findings in this report contravene this conventional wisdom. In this study, the activity of autophagy was increased markedly along with renal fibrosis in Ade-induced renal tubular injury rats, indicating that autophagy was detrimental to renal tubular cells *in vivo*. Initially, we intended to use Ade in the *in vitro* experiments; however, surprisingly we found that Ade could not be dissolved. Thus, HBSS was used to induce autophagy in the *in vitro* study. Besides LC3, beclin1 is another autophagic marker, which usually changes along with LC3. However, HBSS could induce LC3 conversion significantly, but not beclin1 in our study. It is possible that HBSS-induced autophagy observed in NRK cells is beclin1-independent. Further, it is intriguing that rhein alone can induce autophagy, but it exerts anti-autophagic effect as co-treated with HBSS. The same drug may have multiple effects in the physiological and pathological situations. The underling mechanisms need to be explored in the future. Our data clearly showed that rhein could effectively inhibit autophagic activity by regulating the AMPK/mTOR pathways. Unfortunately, no notable apoptosis or cellular morphological changes were observed in NRK-52E cells exposed to HBSS, and therefore, we could not have direct evidence to clarify whether the suppressive effect of rhein on autophagy protected NRK-52E cells from stress-induced cell damage. Further exploration of rhein in different models with renal impairment is urgently needed.

In this study, rhein was found to increase the phosphorylation of mTOR Ser2448 and p70S6K in HBSS-treated NRK-52E cells. The phosphorylation of mTOR Ser2481 was also tested, but it did not change significantly in HBSS stimulation (data not shown). The PI3K/Akt, AMPK and MAPKs signaling pathways have been found to regulate the phosphorylation of mTOR^29^,^35^,^36^. Previous studies also showed that rhein could suppress the activation of PI3K, p-Akt and p-ERK^37^. Cong *et al*. reported that p-Akt/Akt could be diminished by rhein^38^. Interestingly, our data suggested that the PI3K/Akt pathway was not involved in the suppressive effect of rhein on autophagy. In contrast, rhein could attenuate autophagy and activated mTOR signaling via the AMPK-dependent pathway. It is well known that there is a counteracting regulation between Akt and AMPK. However, Akti 10 μM used in this study couldn’t counteract with rhein on Akt activation. Due to the regulation appearing different in cell-type- and condition-dependent, the underling mechanisms need to be explored in the future.

MAPKs are a family of serine/threonine protein kinases involved in a wide range of cellular responses^39^. A series of studies conducted by vom Dahl *et al*. showed that p38 played a key role in cell swelling-induced autophagy, and autophagosome volume decrease was strongly inhibited by colchicine and SB203580^40^. Ponnusamy *et al*. indicated that, necrotic RPTC-Sup induced the activation of all 3 MAPKs pathways in renal fibroblasts^41^, whereas only the inhibition of the Erk pathway could block autophagy^42^. A role for JNK in autophagy has also been studied. Recently Wei *et al*. demonstrated that, during starvation stress, the activation of JNK1 phosphorylated Bcl-2 at multiple sites and lead to its dissociation from beclin1 and induction of autophagy^43^. In this report, we observed that during HBSS-induced starvation, the p38 and Erk pathways, but not the JNK pathway, were activated, and the p38 and Erk pathways could be decreased by rhein. Moreover, p38 and Erk inhibitors, SB203580 and PD098059, respectively, could decrease autophagy induced by HBSS in NRK-52E cells. The role and regulation of individual MAPKs in autophagy are very complex and may vary from one experimental model to another. Both AMPK and p38/Erk MAPKs are important upstream molecules in regulating mTOR activity, through which they regulate autophagy. These molecules may work interdependently in the regulation of autophagy.

In summary, we demonstrated that rhein, a natural autophagic regulator, could inhibit autophagy in rat renal tubular cells by the regulation of the AMPK/mTOR, p38/Erk MAPKs and Akt-independent signaling pathways. These findings may partly explain the therapeutic mechanisms of rhubarb and rhein in treating CKD patients in clinic, and further suggest that targeting autophagy and related signaling pathways may provide new strategies for the treatment of renal fibrosis in CKD.

## Methods

### Reagents

Ade was obtained from Amresco (Solon, OH, USA) and the fresh Ade solution was prepared daily. The 2% Ade was prepared from dissolving 1 g Ade in 50 mL flour solution. Rhubarb granules were purchased from Tianjiang Pharmacology Co. Ltd (Jiangyin, China) and were dissolved in distilled water to a concentration of 1 g/mL for experimental use. HBSS was purchased from HyClone (Logan, Utah, USA). Rhein, emodin, bafilomycin A1, LiCl, rapamycin, insulin, metformin, Akti, PD098059 and SB203580 were obtained from Sigma-Aldrich Chemical Co. (St Louis, MO, USA).

### Animal experiments

Nineteen Sprague-Dawley (SD) male rats, weighing approximately 200 g each, were purchased from the Animal Center of the Nanjing Military District General Hospital (Nanjing, China). The experiments were performed in accordance with protocols approved by the Animal Ethics Committee of Nanjing University Medical School (Permit Number: SCXK (SU) 12014-0001). All rats were housed at 22 ± 3 °C and 50 ± 10% humidity using a 12-hour light/dark cycle and were fed a standard rat chow and given tap water ad libitum in the Experimental Animal Center of The Affiliated Hospital of Nanjing University Medical School. The rats were allowed 1 week to acclimatize before the experiment.

In line with the previous study^8^, we administered 2% Ade at a dose of 150 mg/kg for 2 weeks to generate rats with renal failure. Rats were divided into 3 groups according to the random number table: 5 rats in the Control group (distilled water), 7 rats in the Ade group (Ade + distilled water), and 7 rats in the Ade + Rhubarb group (Ade + rhubarb). Taking the dose of DFD as a reference, 9 g/d rhubarb in this prescription is used to treat a 60 kg patient in clinic. According to the animal standard conversion formula, the effective amount of rhubarb in rats is equivalent to 1 g/kg/d.

Following the administration of Ade for 2 weeks, rhubarb solution was given to the rats in the Ade + Rhubarb group daily by gastric gavage for 3 weeks, while the rats in the Ade and Control groups were treated with 2 mL distilled water in a similar manner. Every 3 days, the rats in the Ade and Ade + Rhubarb groups were given 2% Ade at a total dose of 150 mg/kg to avoid a quick recovery of renal function. At the end of 5 weeks, all rats were anesthetized by intraperitoneal injection of ketamine and diazepam (1:1) and sacrificed by cardiac puncture. The kidneys were collected for the detection of various indicators. The experimental procedure is shown in Table 1.

### Immunohistochemistry

Kidney tissues from the rats were frozen in OCT compound (Sakura Finetek, Tokyo, Japan) and sectioned at a thickness of 4 μm. The cryostat sections were fixed in acetone for 20 minutes at room temperature. Before and after incubating the tissue sections with 0.3% Triton X-100 for 5 minutes, they were washed with phosphate buffered saline (PBS) 3 times. Thereafter, tissue sections were blocked with a blocking solution containing 1% bovine serum albumin. Slides were incubated overnight at 4 °C with primary antibodies against collagen I, fibronectin (Abcam, New Territories, HK) and LC3 I/II (Cell Signaling, Beverly, MA). After washing with PBS 3 times, secondary horseradish peroxidase (HRP)-conjugated anti-rabbit immunoglobulins (Abcam, New Territories, HK) were applied to the slides for 1 hour in the dark at room temperature. After washing with PBS 3 times, the slides were incubated with 3, 3′-diaminobenzidine tetrahydrochloride (DAB) for 5–10 minutes. Using light microscopy, changes in kidneys and the positively stained areas were observed. These positive areas were visualized at a magnification of 200× and the percentages of the positive areas in whole renal areas were calculated in randomly selected 5 nonoverlapping fields with Image-Pro Plus 5.0 software (Media Cybernetics, Silver Spring, MD).

### Cell Culture

NRK-52E cells, a rat renal proximal tubular epithelial cell line, were cultured in Dulbecco’s modified Eagle’s medium/Ham’s F-12 (HyClone) supplemented with 5% fetal bovine serum (FBS; Gibco, Grand Island, NY).

### Western Blot Analysis

Western blot analysis was performed as described before^44^. The level of collagen I and fibronectin were assessed using anti-collagen I antibody and anti-fibronectin antibody (Abcam, New Territories, HK). The levels of LC3 I/II, and phosphorylated proteins of p38, Erk and JNK were assessed using anti-LC3A/B antibody, anti-phospho p38 MAPK (Thr180/Tyr182) (p-p38) antibody, anti-phospho p44/42 MAPK (Erk1/2) (Thr202/Tyr204) (p-Erk) antibody, and anti-phospho SAPK/JNK (Thr183/Tyr185) (p-JNK) antibody (Cell Signaling, Beverly, MA). The levels of the phosphorylated and total proteins of AMPK were assessed using anti-phospho AMPKα (Thr172) and anti-AMPKα antibodies (Cell Signaling, Beverly, MA, USA). The levels of the phosphorylated and total proteins of Akt were assessed using anti-phospho Akt (Ser473) and anti-Akt antibodies (Cell Signaling, Beverly, MA, USA). The levels of the phosphorylated and total proteins of mTOR were assessed using anti-phospho mTOR (Ser2448) and anti-mTOR antibodies (Cell Signaling, Beverly, MA, USA). The level of the phosphorylated protein of p70S6K was assessed using anti-phospho p70S6K (Ser371) antibody (Cell Signaling, Beverly, MA, USA). The level of β-actin was assessed using anti-β-actin antibody (Cell Signaling, Beverly, MA) as a loading control. Blots were visualized using film developer and fixer solutions from Beyotime, Haimen, China. Densitometric analysis was performed using Image J Software.

### Transient Transfection

NRK-52E cells were transiently transfected with pmRFP-LC3, or Deptor, which specifically interacts with mTOR (Addgene, Cambridge, MA) using Lipofectamine 2000 (Invitrogen, Carlsbad, CA, USA) according to the manufacturer’s instructions^45^. NRK-52E cells transfected with Deptor were exposed to insulin or HBSS with or without rhein, and the cells were then collected and subjected to western blot analysis to evaluate the protein expressions of p-p70S6K and LC3 I/II.

### Fluorescence Microscopy

NRK-52E cells transfected with pmRFP-LC3 were exposed to HBSS and bafilomycin A1 with rhein alone or co-treatment of rhein and metformin, or HBSS and bafilomycin A1 with PD098059 or SB203580 for 2 hours. The confocal images were captured at 200× magnification using the Olympus CKX41-F32FL fluorescence microscope (Olympus, Tokyo, Japan).

### Statistical Analysis

Western blot analyses were repeated 2–3 times independently (or performed in triplicate or in quadruplicate), and the individual data were subjected to densitometric analysis. Data were expressed as means ± SD. Statistical analysis was performed using the One-way Analysis of Variance (ANOVA)/non-parametric Mann-Whitney U test (according to normality and homogeneity of variances tests: yes/no) to compare difference among groups. A *P* value < 0.05 was considered to indicate a statistically significant difference.

## Additional Information

**How to cite this article**: Tu, Y. *et al*. Rhein Inhibits Autophagy in Rat Renal Tubular Cells by Regulation of AMPK/mTOR Signaling. *Sci. Rep.* **7**, 43790; doi: 10.1038/srep43790 (2017).

**Publisher's note:** Springer Nature remains neutral with regard to jurisdictional claims in published maps and institutional affiliations.

## Supplementary Material

Supplementary Figure S1

## Figures and Tables

**Table 1:** The experimental procedure of the *in vivo* study.

Group (n)	Experimental procedure (Weeks 0–2)	Experimental procedure (Weeks 3–5)	
		Ante Meridiem	Post Meridiem/3 days
Control (5)	Distilled water 2 mL	Distilled water 2 mL	Distilled water 2 mL
Ade (7)	Ade 150 mg/kg	Distilled water 2 mL	Ade 150 mg/kg
Ade + Rhubarb (7)	Ade 150 mg/kg	Rhubarb 1 g/kg	Ade 150 mg/kg

Abbreviations: Ade, adenine.
