# Supplementary material for: Rhein Inhibits Autophagy in Rat Renal Tubular Cells by Regulation of AMPK/mTOR Signaling
Source: Sci Rep. 2017 Mar 2;7:43790. doi: 10.1038/srep43790 (PMC5333140; doi:10.1038/srep43790)
Supplement: Supplementary Figure S1 [file srep43790-s1.pdf]

# Rhein Inhibits Autophagy in Rat Renal Tubular Cells by Regulation of AMPK/mTOR Signaling

Yue Tu[1, 2, 7], Liubao Gu[3, 7], Diping Chen[2], Wei Wu[4], Hong Liu[5], Hao Hu[6], Yigang Wan[4] & Wei Sun[1]

[1]Department of Nephrology, The Affiliated Hospital of Nanjing University of Chinese Medicine, Nanjing, China; [2] Department of Regimens in Chinese Medicine, Second Clinic Medical School, Nanjing University of Chinese Medicine, Nanjng, China; [3]Center for Diabetes Care, Education and Research, Jiangsu Province Institute of Geriatrics, Nanjing, China; [4]Department of Traditional Chinese Medicine, Nanjing Drum Tower Hospital, The Affiliated Hospital of Nanjing University Medical School, Nanjing, China; [5]First Clinic Medical School, Nanjing University of Chinese Medicine, Nanjng, China; [6]Department of Nephrology, Huanggang Hospital of Traditional Chinese Medicine, Huanggang, China. [7] These authors contributed equally to this work.

Correspondence and requests for materials should be addressed to or Y.W. (email: wyg68918@sina.com) or W.S. (email: 381968515@qq.com)

Running title: Rhein Inhibits Autophagy in Renal Tubular Cells

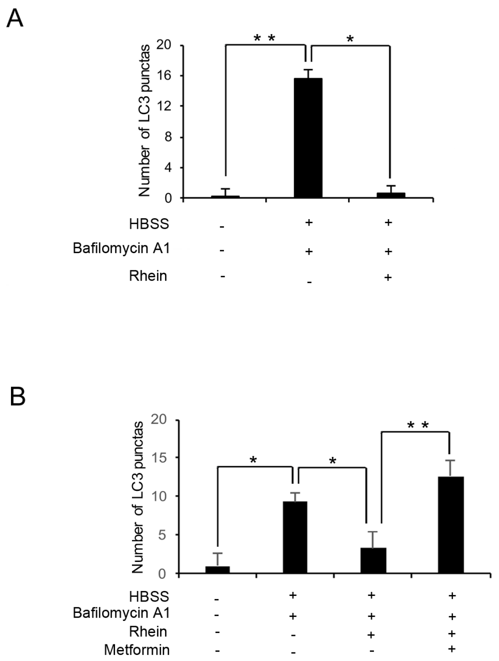


**Figure S1. Quantification of LC3 punctas.** (A) Quantification of LC3 punctas, Related to Figure 2E. (B) Quantification of LC3 punctas, Related to Figure 4F. Data are expressed as mean ± SD, $^{*}P < 0.05$, $^{**}P < 0.01$.
